# Enzymatically Crosslinked Chitosan–Hyaluronic Acid Layer-by-Layer Microcapsules with Controlled Permeability and Enhanced Stability for Cell Encapsulation

**DOI:** 10.3390/polym18091115

**Published:** 2026-04-30

**Authors:** Ririko Terada, Shinji Sakai

**Affiliations:** Department of Materials Engineering Science, Graduate School of Engineering Science, The University of Osaka, Toyonaka 560-8531, Osaka, Japan; terada.ri@cheng.es.osaka-u.ac.jp

**Keywords:** microcapsule, cell therapy, covalent crosslinking, horseradish peroxidase

## Abstract

Cell encapsulation within semipermeable membranes is a promising strategy for protecting transplanted cells from host immune responses, while permitting the diffusion of nutrients and therapeutic molecules. Although alginate-based microcapsules are commonly used, ionically crosslinked capsules often exhibit limited structural stability and tunability in terms of membrane permeability. In this study, we developed covalently stabilized microcapsules. Alginate microgel beads were first prepared as sacrificial templates and subsequently coated with phenol-modified chitosan and hyaluronic acid (Chitosan–Ph and HA-Ph) via layer-by-layer assembly. The multilayer membrane was then covalently stabilized through horseradish peroxidase (HRP)-mediated oxidative coupling of phenol groups, followed by liquefaction of the alginate core. The crosslinked microcapsules maintained structural integrity after liquefaction, while markedly reducing γ-globulin permeation under in vitro conditions and preserving β-cell viability and glucose responsiveness. The findings of this study demonstrate the feasibility of this system as an in vitro platform for stable cell encapsulation, with potential relevance to cell therapy.

## 1. Introduction

Cell encapsulation within semipermeable membranes has been widely investigated to protect transplanted cells from host immune responses, while enabling the exchange of oxygen, nutrients, and therapeutic molecules [1,2,3]. This approach is particularly promising for cell-based therapies using insulin-producing cells [4], as encapsulation may improve graft survival and function without the need for systemic immunosuppression [5,6]. Among the various systems, hydrogel-based microcapsules are of particular interest because they provide a cell-compatible microenvironment [7,8]. In particular, layer-by-layer (LBL) assembly of oppositely charged polyelectrolytes on Ca-alginate microgel beads has been widely used to form semipermeable multilayer membranes [9]. In this system, the core Ca-alginate microgel is liquefied after the formation of a semipermeable membrane to enhance oxygen and nutrient supply to the enclosed cells [10]. However, this process often produces microcapsules with insufficient stability [11,12], which may impair immune isolation and reduce the survival of encapsulated cells [13]. Introducing covalent crosslinking within the capsule membrane may improve structural stability.

Several covalent crosslinking strategies have been proposed [14,15]. For example, carbodiimide crosslinking and photopolymerization of methacrylated polymers have been used to reinforce microcapsules [16,17]. These approaches can improve mechanical strength and structural integrity and have been investigated for cell encapsulation applications [18,19,20]. However, crosslinking processes often require reactive chemicals or photoinitiators, which may compromise cell viability and function [21,22].

Phenol-modified polymers enable mild, aqueous, and cell-compatible covalent crosslinking under physiological conditions [23,24,25]. Among them, phenol-modified natural polymers are attractive building blocks for cell-encapsulation systems because they can be crosslinked without harsh initiators or organic reagents and have been used as biocompatible hydrogel-forming materials [26,27]. In particular, the combination of cationic chitosan and anionic hyaluronic acid is advantageous because these oppositely charged natural polymers can form multilayer membranes by LBL assembly while also permitting subsequent enzymatic covalent stabilization. In addition, hyaluronic acid supports the physiological functions and survival of various cell types, including stem cells [28].

**Figure 1:**
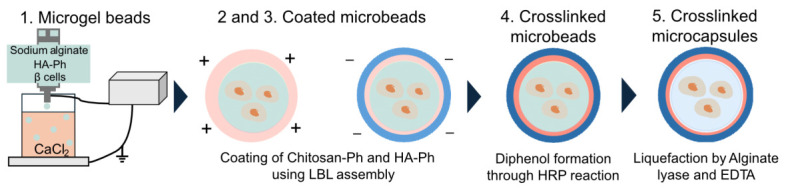
Schematic diagram of crosslinked microcapsule fabrication. Green, pink and blue represent alginate, Chitosan-Ph and HA-Ph, respectively.

In this study, we aimed to develop microcapsules with LBL-fabricated membranes that were covalently stabilized via horseradish peroxidase (HRP)-mediated crosslinking of phenol-modified chitosan (Chitosan–Ph) and phenol-modified hyaluronic acid (HA-Ph) for cell encapsulation (Figure 1). Ca-alginate microgel beads were prepared as sacrificial templates and coated with phenol-modified polymers via LBL assembly. The membrane was then stabilized by HRP-mediated oxidative coupling of phenol groups, followed by liquefaction of the alginate core. Unlike other HRP-crosslinked microbeads composed of single polymer systems without multilayer membranes [29,30], the present approach enables enzymatic covalent stabilization of LBL membranes. We hypothesized that this strategy would improve capsule stability without compromising the permeability and cytocompatibility required for cell encapsulation. To test this hypothesis, we evaluated the microcapsules in terms of immunoisolation-related barrier function by examining permeability to IgG-sized molecules using γ-globulin as a model macromolecule, and assessed their suitability for cell encapsulation based on β-cell viability and glucose-responsive insulin secretion.

## 2. Materials and Methods

### 2.1. Materials

Chitosan (Mw 50,000–100,000, product code: LL-40), sodium alginate (Mw 70,000, product code: I-1G), and hyaluronic acid (Na-HA, Mw 550,000, product code: HA-LQ) were obtained from Yaizu Suisankagaku Industries (Osaka, Japan), Kimica (Tokyo, Japan), and Kewpie (Tokyo, Japan), respectively. N-hydroxysuccinimide (NHS, for peptide synthesis, 98.0–102.0% by titration), HRP (for biochemistry; 140 U/mg), 5-aminofluorescein (AF), Rhodamine B (RB, guaranteed reagent), N,N,N′,N′-tetramethyl-ethylenediamine (98.0+%, capillary GC), 3-(4-hydroxyphenyl)-propionic acid(HPP), and hydrogen peroxide (H_2_O_2_) [30% (*w*/*w*)] were purchased from Fujifilm Wako Pure Chemical Industries (Osaka, Japan). Propidium iodide (PI) was purchased from Dojindo Laboratories (Kumamoto, Japan). Calcein-AM (≥90.0% on HPLC) and coelenterazine (>98% on HPLC) were purchased from Nacalai Tesque (Kyoto, Japan). Water-soluble carbodiimide (EDC) and poly-L-lysine (MWCO 12,000) were purchased from the Peptide Institute (Osaka, Japan). Tyramine hydrochloride (>98.0% by titration) and yatalase were purchased from Tokyo Chemical Industry (Tokyo, Japan) and Takara Bio (Shiga, Japan), respectively. All chemicals were used as received unless otherwise noted.

### 2.2. Synthesis of Chitosan–Ph and HA-Ph

Chitosan–Ph and HA-Ph were prepared by conjugating tyramine to the polymer backbone via carbodiimide-mediated coupling, as previously reported [26,27].

Briefly, chitosan was dissolved at 3.0% (*w*/*v*) in 20 mM HCl containing 1% (*v*/*v*)N, N, N’, N’-tetramethylethylenediamine, and the pH was adjusted to 4.6. Then, 0.032% (*w*/*v*) lactobionic acid, 0.12% (*w*/*v*) HPP, and 0.23% (*w*/*v*) EDC were added sequentially, and the mixture was stirred for 20 h. The reaction solution was dialyzed against deionized water using a dialysis membrane (MWCO: 12,000–14,000) to remove unreacted materials. This process was continued until no absorbance peak was observed in the supernatant at ~275 nm, corresponding to the phenol group. After freeze-drying, the samples were stored at 4 °C. The phenol content was 3.69 × 10^−5^ mol/g Chitosan–Ph, determined from absorbance at 275 nm using 0.01 M HCl containing 0.1% (*w*/*v*) chitosan or Chitosan–Ph.

Na-HA was dissolved in 0.1 M MES buffer solution at 0.75% (*w*/*v*), and the pH was adjusted to 6.0. Further, 1.21% (*w*/*v*) tyramine, 0.314% (*w*/*v*) NHS, and 0.552% (*w*/*v*) EDC were sequentially dissolved and stirred for 19 h. Unreacted components were removed by dialysis, and the mixture was freeze-dried. The phenol content was 3.36 × 10^−4^ mol/g HA-Ph, determined using 0.1% (*w*/*v*) Na-HA or HA-Ph in Milli-Q ultrapure water. The HA-Ph solution was sonicated for 30 min at 50 °C before use.

### 2.3. Fabrication of Crosslinked Microcapsules

#### 2.3.1. Preparation

Calcium-free Krebs–Ringer HEPES-buffered (CF-KRH) solution (pH 7.4) containing 1.5% (*w*/*v*) sodium alginate and 0.5% (*w*/*v*) HA-Ph was dispensed at 5.0 mL/h from a 27-G stainless steel needle into 10 mM HEPES solution (pH 7.4) containing 50 mM CaCl_2_ using an electrostatic droplet device. A 10 kV voltage was applied to the needle, and the distance between the solution and needle was set to 10 cm. After rinsing with the CF-KRH solution, the microgel beads were immersed in CF-KRH solution (pH 6.7) containing 1.0% (*w*/*v*) Chitosan–Ph for 10 min and rinsed with CaCl_2_ solution. The obtained microbeads were immersed in CaCl_2_ solution (pH 7.4) containing 0.1% (*w*/*v*) HA-Ph for 10 min and rinsed with KRH solution. The coated microbeads were immersed in KRH solution (pH 7.4) containing 1 U/mL HRP and 0.1 mM H_2_O_2_ for 10 min, and then rinsed with KRH solution. The crosslinked microbeads were incubated in KRH solution (pH 7.4) containing 0.25 mg/mL alginate lyase at 37 °C for 60 min and then stirred in phosphate-buffered saline (PBS) containing 5 mM EDTA for 5 min. Finally, the crosslinked microcapsules were washed with PBS.

#### 2.3.2. Layer-by-Layer Coating of Microbeads

To evaluate the applicability of the LBL assembly to HA-Ph and Chitosan–Ph, the microgel beads were soaked in solutions containing fluorescently labeled polymers (Section 2.3.1). The coated microbeads were suspended in CaCl_2_ solution and observed under a fluorescence microscope (APX100, Evident, Nagano, Japan). The synthesis of RB–Chitosan–Ph and FITC-HA-Ph are described in the Supplementary Materials [31]. Fluorescent labeling was used to distinguish each polymer component and to visualize multilayer formation during LBL assembly.

#### 2.3.3. HRP-Mediated Crosslinking of Coated Microbeads

To evaluate the effect of H_2_O_2_ on HRP-mediated diphenol formation, the microcapsules were prepared using 0–1 mM H_2_O_2_ and 1 U/mL HRP (Section 2.3.1). The crosslinked microcapsules were suspended in PBS and observed under the fluorescence microscope. Diphenol formation was evaluated by dividing the fluorescence intensity within the microcapsules by that outside the capsules.

#### 2.3.4. Liquefaction of Alginate Microgel Through Ca^2+^ Chelation and Alginate Degradation

The liquefaction of Ca-alginate microgels was investigated based on Ca^2+^ chelation and enzymatic degradation. Crosslinked microbeads were prepared using AF-alginate (Section 2.3.1), which emits green fluorescence, to visually confirm the liquefaction of alginate microgels and were immersed in 10 mM HEPES solution (pH 7.4) containing 55 mM citrate or KRH solution (pH 7.4) containing alginate lyase. Changes in the diameter and fluorescence intensity were observed using a fluorescence microscope (BZ-X800L, Keyence, Osaka, Japan). The synthesis of AF-alginate is described in the Supplementary Materials.

### 2.4. Stability of Crosslinked Microcapsules

To evaluate the ability of the crosslinked microcapsules to maintain their shape under mechanical stress and in the presence of enzymes, osmotic pressure and enzymatic degradation tests were conducted.

#### 2.4.1. Preparation of Alginate-Poly-L-Lysine-Alginate (APA) Microcapsules

The APA microcapsules were fabricated as previously reported [32]. To coat the microgel beads (Section 2.3.1) with poly-L-lysine and alginate, the microbeads were soaked in CF-KRH solution (pH 7.4) containing 0.05% (*w*/*v*) poly-L-lysine for 5 min and in CF-KRH solution (pH 7.4) containing 0.18% (*w*/*v*) alginate for 4 min after rinsing with CF-KRH solution (pH 7.4). To liquefy the Ca-alginate microgel, the coated microbeads were stirred in 10 mM HEPES solution (pH 7.4) containing 55 mM sodium citrate for 10 min. APA microcapsules were rinsed with PBS.

#### 2.4.2. Osmotic Pressure Test

The osmotic pressure test was conducted according to the previously reported method [33]. The microcapsules were incubated in ultrapure water (H_2_O, 0 mOsm/kg) at 37 °C for 2 h. The solution was replaced with PBS (276–284 mOsm/kg, pH 7.4), and the microcapsules were incubated up to 7 d. The intact capsule ratio, swelling ratio, and recovery rate were expressed as follows:

(1) $$Intact\;capsule\;ratio\;\left(-\right)=\;\frac{N_{All}-\;N_{c}}{N_{All}},$$

(2) $$Swelling\;ratio\;(-)=\frac{D_{H_{2}O}}{D_{PBS}},$$

(3) $$Recovery\;ratio\left(-\right)=\frac{\left(D_{day\;0}-D_{PBS}\right)}{\left(D_{H_{2}O}-D_{PBS}\right)},$$

where $N_{All}$, $N_{c}$, $D_{PBS}$, $D_{H_{2}O}$, and $D_{day0}$ are the number of all capsules, number of crushed capsules, and diameter of microcapsules in PBS, in H_2_O, and on day 0, respectively.

#### 2.4.3. Enzyme Resistance Test

The microcapsules were incubated at 37 °C in PBS containing 0.165 mg/mL hyaluronidase or yatalase. The intact capsule ratio was calculated using Formula (1).

### 2.5. Permeability of Microcapsules

#### 2.5.1. Permeability for Antibody and Protein

The permeability of the microcapsules for antibodies was evaluated based on the diffusion of FITC-γ-globulin. The microcapsules (Section 2.3.1) were immersed in a FITC-γ-globulin or FITC-bovine serum albumin (BSA) solution, and observed under a fluorescence microscope for 120 min. The diffusion of FITC-γ-globulin and FITC-BSA was evaluated based on the fluorescence intensity within divided by the fluorescence intensity outside the microcapsule. The synthesis of FITC-γ-globulin and FITC-BSA is described in the Supplementary Materials.

#### 2.5.2. Release of Insulin-like Molecules from Crosslinked Microcapsules

The release profile of insulin-like molecules from crosslinked microcapsules was evaluated based on the diffusion of AF-grafted biocompatible anchor for cell membrane (BAM). After overnight immersion in an AF-BAM solution, the microcapsules (Section 2.3.1) were dispersed in PBS and observed under a fluorescence microscope for 60 min. The release of AF-BAM was evaluated by the fluorescence intensity inside the capsules compared to that at 0 min. Details regarding the synthesis of AF-BAM are provided in the Supplementary Materials.

### 2.6. Encapsulation of β-Cells into the Crosslinked Microcapsules

To evaluate the cytocompatibility and applicability to cell therapy, commercially available iGL cells (rat pancreatic β-cell line, Cosmo Bio, Tokyo, Japan) were encapsulated within the crosslinked microcapsules.

#### 2.6.1. β-Cell Encapsulation

The microcapsules were fabricated using CF-KRH solution (pH 7.4) containing 1.5% (*w*/*v*) alginate, 0.5% (*w*/*v*) HA-Ph, and 4.0 × 10^6^ cells/mL iGL cells as described in Section 2.3.1. After HRP-mediated crosslinking, the microbeads were treated with alginate lyase and EDTA to obtain cell-encapsulating crosslinked microcapsules.

#### 2.6.2. Cytocompatibility of Crosslinked Microcapsules

Fluorescent live/dead staining with Calcein-AM and PI was performed at each fabrication step. Additionally, the cell-enclosed microcapsules were dispersed in the medium and transferred to a 24-well or 12-well plate. iGL cells were seeded at 1.0 × 10^5^ cells/mL in the 12-well plate. Cell viability and mitochondrial activity were assessed using fluorescent live/dead staining and a Cell Counting Kit-8 (Dojindo, Kumamoto, Japan).

#### 2.6.3. Insulin Secretion from Encapsulated β-Cells

Encapsulated iGL and naked cells cultured for 4 d were washed twice with KRH solution (pH 7.4) containing 0.01 *w*/*v*% BSA (BSA-KRH) and incubated in BSA-KRH for 1 h to remove residual glucose. The samples were incubated in 20 mM glucose-containing BSA-KRH (G-BSA-KRH) for 1 h, after which 50 µL of the supernatant was added to 200 µL PBS containing 0.01% (*w*/*v*) coelenterazine, and the luminescence intensity at 488 nm was measured [34]. The stimulation index was calculated as follows:

(4) $$Stimulation\;index\;\left(SI\right)=\;\frac{Luminescence\;intensity\;in\;G-BSA-KRH}{Luminescence\;intensity\;in\;BSA-KRH}.$$

### 2.7. Statistical Analysis

Microsoft Excel was used for data analysis and graphing. All quantitative data are presented as mean ± standard deviation (S.D.). For comparisons between two groups, Student’s *t*-test was used. For comparisons among three or more groups, one-way analysis of variance (ANOVA) followed by an appropriate post hoc test was used [35]. Unless otherwise stated, the *n* values shown in the figures indicate the number of individual capsules or wells analyzed per condition. For fluorescence-based analyses, capsules from a single preparation batch were treated as technical replicates. For particle-counting analyses, including capsule diameter distribution and intact capsule ratio, the number of analyzed capsules is indicated in the corresponding figure legends.

### 2.8. Hygienic Handling and Disposal

All cell-related experiments were performed using aseptic techniques, and experimental materials were handled and disposed of in accordance with the regulations of The University of Osaka.

## 3. Results

### 3.1. Fabrication of HA-Ph/Chitosan–Ph Crosslinked Microcapsules

**Figure 2:**
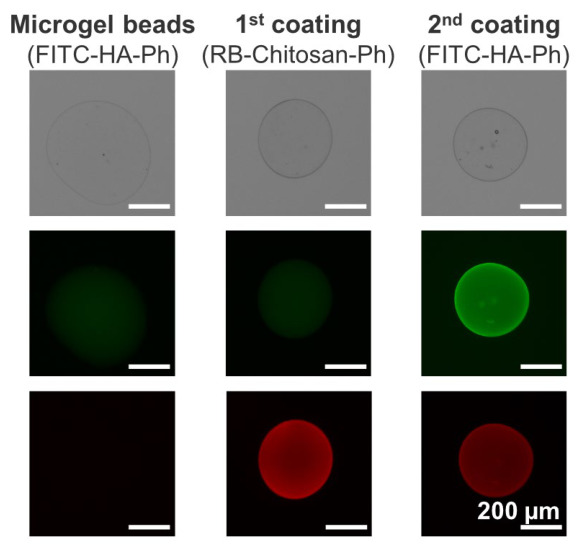
Fluorescence images of microgel beads prepared by dropping HA-Ph and alginate solution into CaCl_2_ solution, followed by sequential immersion in Chitosan–Ph solution (1st coating) and HA-Ph solution (2nd coating). Green and red fluorescence represent FITC-HA-Ph and RB–Chitosan–Ph, respectively.

Figure 2 shows the phenol-grafted polymers layered onto the microgel beads via LBL assembly. Strong green fluorescence was observed in the microgel beads, indicating that HA-Ph was enclosed within the Ca-alginate microgel. The microbeads coated with rhodamine B (RB)-grafted Chitosan–Ph and FITC-grafted HA-Ph exhibited localization of Chitosan–Ph and HA-Ph near the surface. These results indicate the feasibility of coating beads with Chitosan–Ph and HA-Ph via LBL assembly.

**Figure 3:**
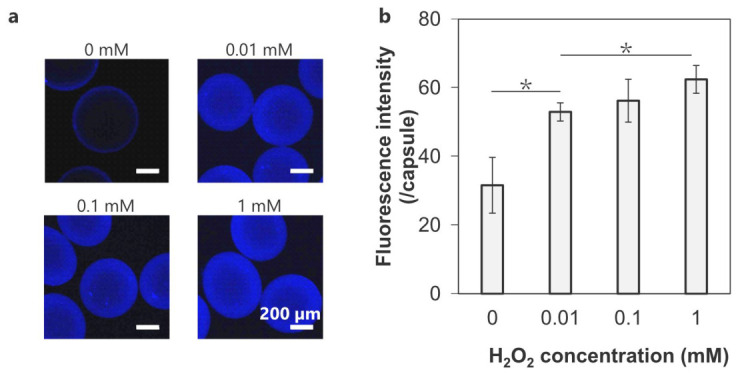
(**a**) Fluorescence images of the microcapsules crosslinked through HRP-mediated reaction at 0–1 mM H_2_O_2_. Blue fluorescence indicates diphenol formation. (**b**) Fluorescence intensity within each crosslinked microcapsule (*n* = 5 capsules from one representative batch; error bars: S.D. * *p* < 0.05).

HRP-mediated diphenol formation in the crosslinked microcapsules was visualized using blue fluorescence (Figure 3). The strong blue fluorescence on the outer layer of the capsules indicated a higher crosslinking density resulting from denser contact between Chitosan–Ph and HA-Ph in the coating than between HA-Ph on the inner side of the microbeads (Figure 3a). The non-crosslinked microcapsules (0 mM) exhibited the lowest fluorescence intensity (32 ± 8), whereas the microcapsules crosslinked at 0.01, 0.1, and 1 mM H_2_O_2_ showed fluorescence intensities of 53 ± 3, 56 ± 6, and 62 ± 4, respectively (Figure 3b). Although there was no significant difference between the specimens at 0.1 and 1 mM, the fluorescence intensity increased with increasing H_2_O_2_ concentration. This demonstrates that crosslinking density can be controlled by adjusting H_2_O_2_ concentration, a characteristic necessary for balancing the permeability and stability of microcapsules.

**Figure 4:**
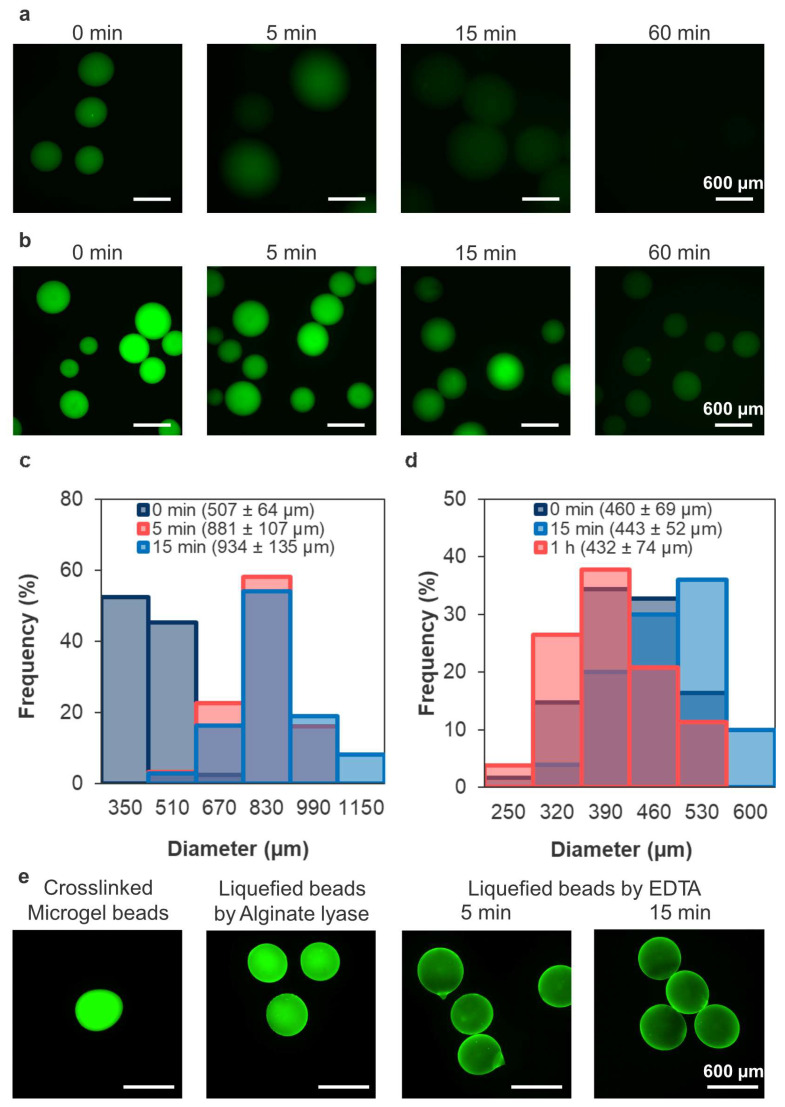
Fluorescence images of the crosslinked microcapsules prepared using AF-alginate in solutions containing (**a**) citric acid or (**b**) alginate lyase. Green fluorescence shows AF-alginate. Change in diameter of the crosslinked microbeads after immersion in a solution containing (**c**) citric acid or (**d**) alginate lyase (*n* > 30 capsules from one representative batch). (**e**) Fluorescence images of the microbeads exhibiting a green fluorescence from alginate that gradually fades over time due to alginate lyase and EDTA.

The crosslinked microbeads prepared using 5-aminofluorescein (AF)-grafted alginate and soaked in a solution containing citric acid or alginate lyase revealed the effect of the liquefaction method on the liquefaction rate and capsule morphology (Figure 4). The fading fluorescence revealed that sodium citrate liquefied the alginate microgel faster than alginate lyase (Figure 4a,b). After 15 min of immersion, the diameter increased from 507 ± 64 µm to 934 ± 135 µm for the microbeads placed in sodium citrate solution, and from 460 ± 69 µm to 443 ± 52 µm for those placed in a solution containing alginate lyase (Figure 4c,d). These results suggest that the method affected both liquefaction speed and maintenance of capsule morphology.

We hypothesized that combining Ca^2+^ chelation with enzymatic alginate degradation would enable degraded alginate to diffuse out more easily, thereby liquefying the alginate microgel effectively without significantly altering capsule diameter. The crosslinked microbeads incubated in alginate lyase solution exhibited weakened green fluorescence, which diminished further upon immersion in EDTA solution within 5 min (Figure 4e). These results suggest that combining alginate lyase and EDTA enables liquefaction of alginate microgels without notable morphological changes.

### 3.2. Stability of Crosslinked Microcapsules

**Figure 5:**
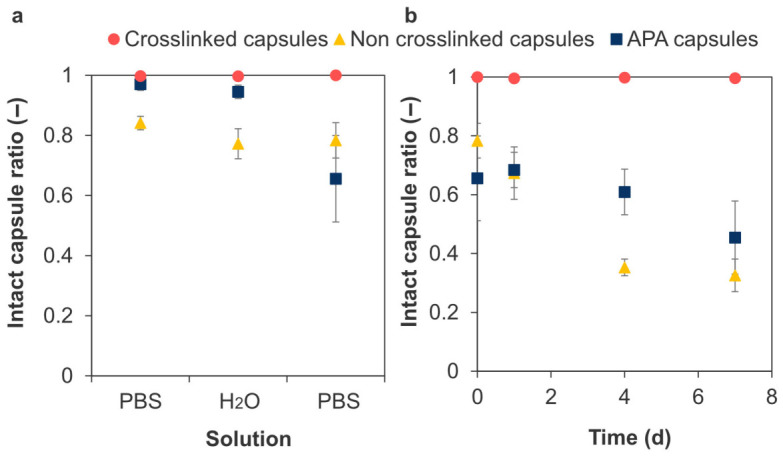
Intact capsule ratio in osmotic pressure tests. Effect of (**a**) osmotic pressure change with sequential immersion in PBS in ultrapure water (H_2_O) for 2 h and in PBS for 15 min. (**b**) Effect of incubation time in PBS. The value at day 0 corresponds to the capsules immediately after the sequential immersion test in (**a**) (*n* > 180 capsules per preparation, 3 independent preparations; error bars: S.D.).

The effect of covalent crosslinking on microcapsule stability was evaluated using osmotic pressure and enzyme degradation tests. The crosslinked microcapsules exhibited superior resistance to changes in osmotic pressure compared to the non-crosslinked and alginate-poly-L-lysine-alginate (APA) microcapsules (Figure 5a). The crosslinked microcapsules maintained their spherical shape even after 7 d of incubation at 37 °C in PBS, with the intact capsule ratio exceeding 0.99 (Figure 5b). In contrast, the non-crosslinked microcapsules and APA microcapsules showed structural disruption over time, with the ratio decreasing to 0.33 ± 0.06 and 0.45 ± 0.12 by day 7, respectively. These results suggest that the introduction of covalent bonds enhanced the stability of microcapsules.

**Figure 6:**
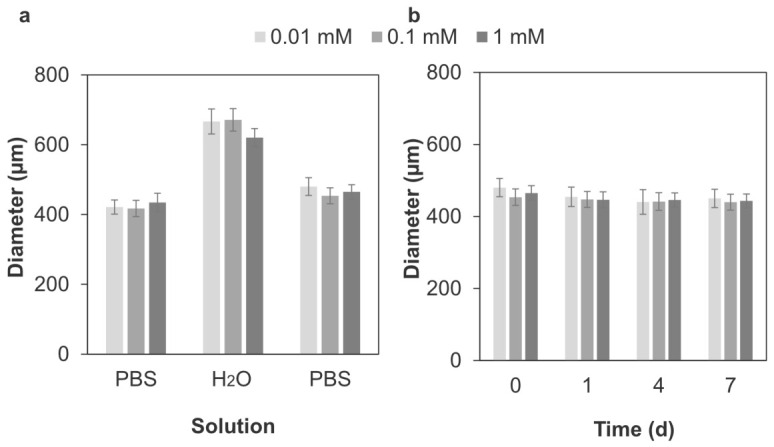
Effect of H_2_O_2_ concentration during HRP-mediated crosslinking on microcapsule diameter in osmotic pressure test (*n* > 100 capsules from one representative batch, error bars: S.D.). The microcapsules were (**a**) sequentially immersed in PBS, in ultrapure water (H_2_O) for 2 h, and in PBS for 15 min, and (**b**) immersed in PBS for 7 d.

The effect of H_2_O_2_ concentration on capsule stability was reflected in the change in capsule diameter during osmotic pressure testing (Figure 6). The crosslinked microcapsules swelled in ultrapure water (H_2_O), recovered in PBS (Figure 6a), and showed no significant change in diameter after 7 d (Figure 6b). The intact capsule ratio for the microcapsules prepared at 0.01 mM was slightly lower (>0.94) than that for the crosslinked microcapsules prepared at 0.1 mM and 1 mM (>0.99) throughout this experiment. Although no significant difference was observed, the swelling ratio showed a decreasing trend as H_2_O_2_ concentration increased (Table 1). The recovery ratio for 1 mM and 0.1 mM were nearly identical (0.837 ± 0.003 vs. 0.847 ± 0.003) and higher than that for 0.01 mM (0.765 ± 0.003; Table 1). These results suggest that H_2_O_2_ concentration affects the stability of microcapsules. Therefore, the crosslinked microcapsules prepared with 0.1 mM H_2_O_2_ were used in subsequent experiments.

**Figure 7:**
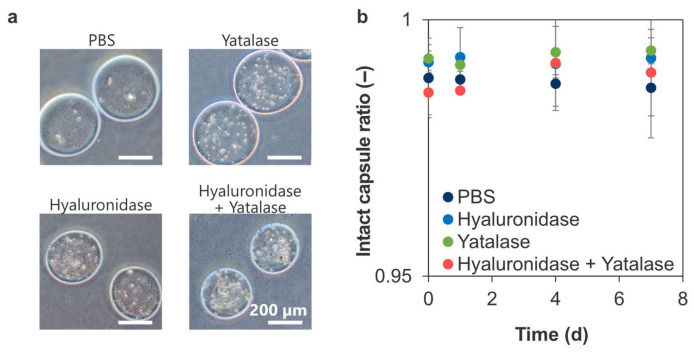
(**a**) Photographs of crosslinked microcapsules incubated in PBS containing hyaluronidase and yatalase. (**b**) Intact capsule ratio for crosslinked microcapsules immersed in enzyme solutions (*n* > 200 capsules per preparation, 3 independent preparations; error bars: S.D.).

Figure 7a shows that incubation with hyaluronidase caused the capsules to contract, whereas incubation with yatalase caused the capsules to swell while maintaining their spherical shape for 7 d. The intact capsule ratio was similar for the capsules in PBS (0.99 ± 0.01) and those incubated in solutions containing hyaluronidase or yatalase (0.993 ± 0.001 and 0.994 ± 0.007, respectively: Figure 7b). Therefore, covalent bonding improves the stability of microcapsules against polymer-degrading enzymes. However, its effect is insufficient to completely alter the enzymatic degradability of the polymer.

### 3.3. Permeability into Microcapsules

**Figure 8:**
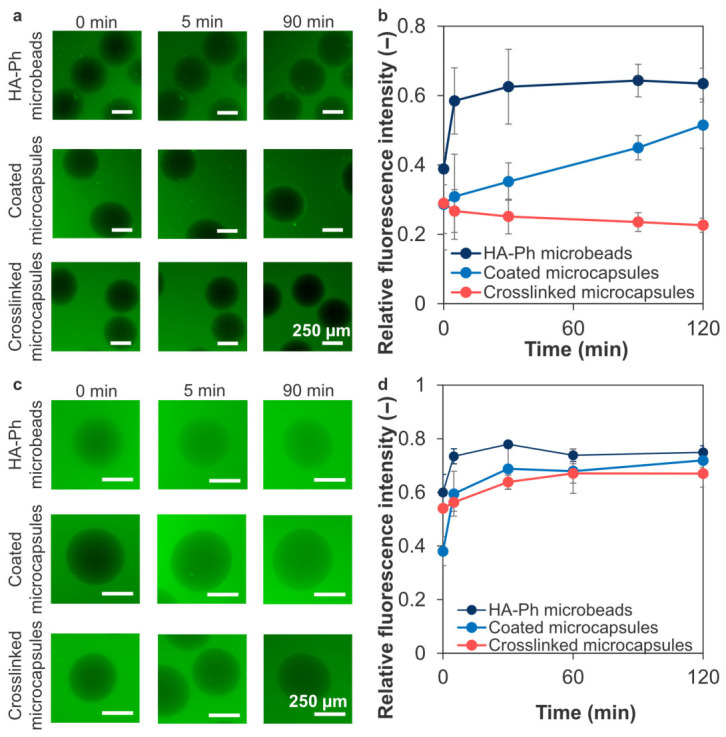
(**a**,**c**) Representative fluorescence micrographs showing diffusion of FITC-γ-globulin and FITC-BSA from the external solution into the HA-Ph microbeads, LBL-coated microcapsules without HRP-mediated crosslinking (coated microcapsules), and LBL-coated microcapsules with HRP-mediated crosslinking (crosslinked microcapsules). (**b**,**d**) Time-dependent change in fluorescence intensity inside the microbeads and microcapsules (*n* = 3–6 capsules from one representative batch per condition, error bars: S.D.).

Figure 8a shows the diffusion of FITC-γ-globulin into microcapsules. In the HA-Ph microbeads, FITC-γ-globulin reached equilibrium within 60–90 min of immersion (Figure 8b). The fluorescence intensity in the coated microcapsules increased over time from 0.3 ± 0.1 at 0 min to 0.51 ± 0.07 at 120 min, indicating that the diffusion of γ-globulin was delayed by coating. The intensity of the crosslinked microcapsules remained at ~0.25 for 120 min, indicating that γ-globulin diffusion was markedly reduced over the observation period. In contrast, FITC-BSA diffused into the capsules (Figure 8c). The fluorescence intensity increased over time, particularly between 0 and 5 min, and reached equilibrium in the following order: HA-Ph microbeads, coated microcapsules and crosslinked microcapsules (Figure 8d). Therefore, coating and subsequent covalent stabilization appear to be effective for reducing the penetration of larger proteins into the microcapsules under the present in vitro conditions.

**Figure 9:**
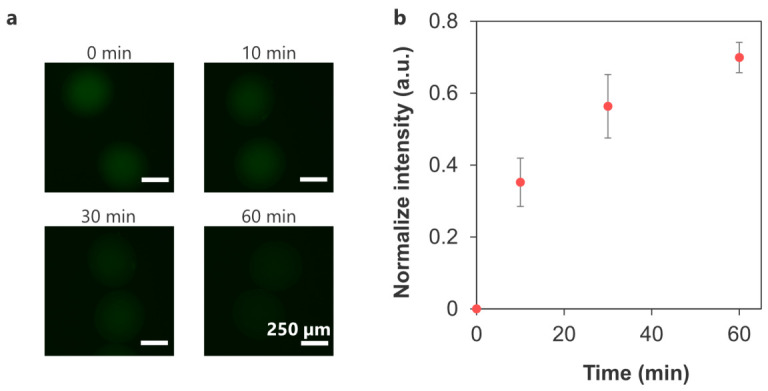
(**a**) Representative fluorescence micrographs showing release of AF-BAM from the crosslinked microcapsules. (**b**) Time-dependent change in fluorescence intensity of the crosslinked microcapsules normalized to that at 0 min (*n* = 10 capsules from one representative batch, error bars: S.D.).

Figure 9a shows the AF-BAM release profile of the crosslinked microcapsules. The fluorescence intensity increased over time to 0.70 ± 0.04 at 60 min, indicating that the AF-BAM diffused out from the crosslinked microcapsules (Figure 9b). Therefore, crosslinked microcapsules may not significantly inhibit the diffusion of molecules with a size comparable to BAM (PEG MW 8000) under the present experimental conditions.

### 3.4. Encapsulation of β-Cells

**Figure 10:**
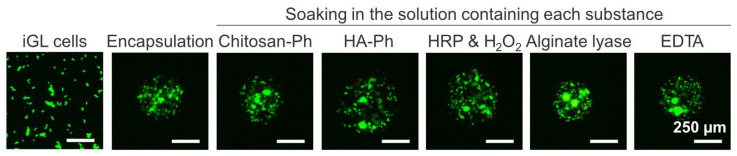
Live/dead staining images of iGL cells during encapsulation in the crosslinked microcapsules. Cell viability was evaluated after soaking in each solution used in the fabrication process (Chitosan–Ph, HA-Ph, HRP & H_2_O_2_, alginate lyase, and EDTA). Green and red fluorescence indicate live and dead cells, respectively.

Figure 10 shows that iGL cells (pancreatic β-cells) exposed to each microcapsule fabrication step remained viable, with no significant increase in dead cells. This indicated that the fabrication process does not significantly damage the cells.

**Figure 11:**
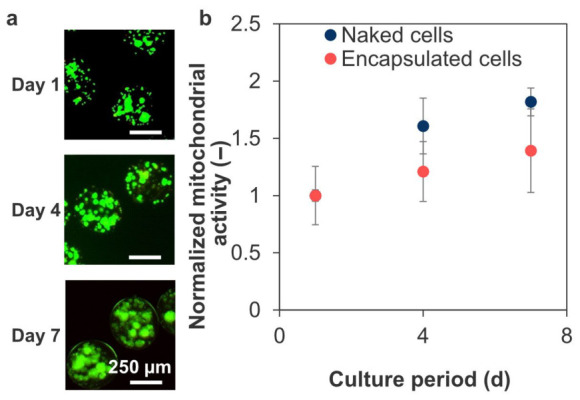
(**a**) Live/dead staining images of encapsulated iGL cells in the crosslinked microcapsules stained at 1, 4, and 7 d after encapsulation. (**b**) Mitochondrial activity of encapsulated and naked iGL cells normalized to that at day 1 measured using WST-8 assay (*n* = 4 wells from one representative batch; error bars: S.D.). Mitochondrial activity of encapsulated and naked cells at day 1 were 6.3 ± 1.1 × 10^−4/^microcapsule and 0.219 ± 0.007/well, respectively.

The encapsulated cells remained viable after 7 d of culture (Figure 11a). During this period, mitochondrial activity increased ~1.4-fold compared to that observed 1 d after encapsulation (Figure 11b), corresponding to a ~70% increase over that observed in naked cell culture.

**Figure 12:**
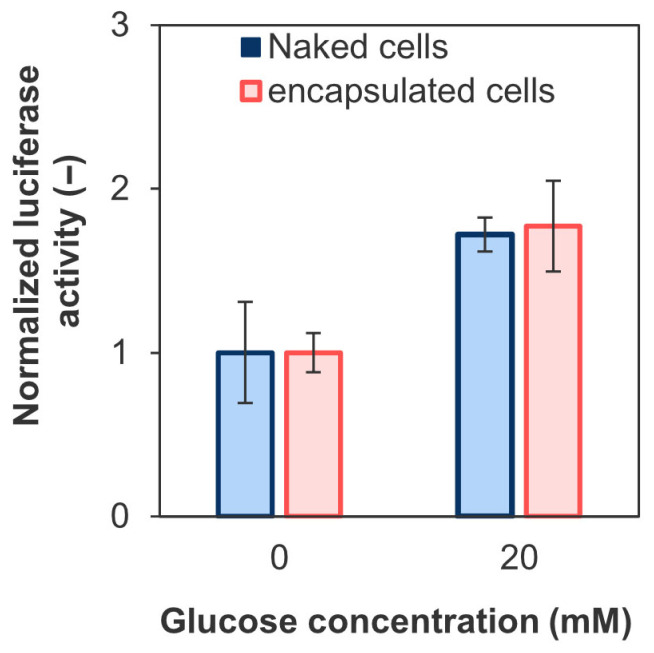
Insulin secretion from naked and encapsulated iGL cells stimulated by BSA-KRH (glucose concentration: 0 mM) and G-BSA-KRH (20 mM) normalized to that at day 1 evaluated based on luciferase activity measured by coelenterazine luminescence at 488 nm (*n* = 3, 4 wells from one representative batch; error bars: S.D.). Luciferase activity values at day 1 of naked and encapsulated iGL cells were 31 ± 10 rlu/well and 0.21 ± 0.02 rlu/capsule, respectively.

Figure 12 shows the changes in insulin secretion by the encapsulated and naked cells in response to increased glucose concentrations. The encapsulated cells increased insulin secretion by 1.8 ± 0.3-fold in a solution containing 20 mM glucose, which mimics hyperglycemia, compared with a glucose-free solution. These results indicate that encapsulated iGL cells retained glucose responsiveness under the tested in vitro conditions. However, the present static glucose-stimulation assay does not directly resolve detailed secretion dynamics such as first- and second-phase insulin release. More detailed functional analyses, including dynamic perfusion or time-resolved imaging approaches, will be necessary in future studies.

## 4. Discussion

This study demonstrated the successful fabrication of microcapsules with covalently crosslinked membranes through LBL assembly on microbeads, HRP-mediated crosslinking of the membranes, and subsequent liquefaction of the alginate hydrogel core. The resulting microcapsules exhibited improved structural stability, resistance to enzyme-induced degradation under the tested in vitro conditions, and reduced permeability to γ-globulin-sized molecules. Furthermore, they showed cytocompatibility with β-cells, which maintained glucose-responsive insulin secretion.

### 4.1. Fabrication of HA-Ph/Chitosan–Ph Crosslinked Microcapsules

LBL assembly is a technique for coating capsules with negatively or positively charged polymers via electrostatic interactions. Chitosan and HA are positively and negatively charged, respectively [36,37]. Since these charges were not reversed following phenol group modification, it was possible to coat the microgel beads via LBL assembly (Figure 2). However, the charge may decrease because the carboxyl and amino groups, which dominate the polymer charge, are modified [38], resulting in easily deformed coated microcapsules (Figure 5).

H_2_O_2_ concentration-dependent diphenol formation in the microcapsules (Figure 3) is consistent with the behavior observed in phenol-grafted polymer-based hydrogels [39]. This is because the probability of diphenol formation varies according to the H_2_O_2_ concentration, which acts as a substrate in the HRP-mediated reaction [40]. Although a higher crosslinking density increases microcapsule stability, it also limits the permeability of biomolecules [41,42]. Additionally, H_2_O_2_ can induce oxidative stress, leading to apoptosis and loss of cellular function [43,44]. Thus, the H_2_O_2_ concentration must be adjusted to fabricate highly stable microcapsules under mild conditions.

Liquefaction of the alginate microgel through Ca^2+^ chelation and enzymatic degradation demonstrated the suitable permeability of the crosslinked microcapsules (Figure 4). The pore size of the microcapsules was larger than that of citric acid (MW 192), suggesting that low-molecular-weight substances can pass through. This is crucial for cell survival, as it enables the diffusion of essential substances such as glucose (MW 180), amino acids (MW ~hundreds), and oxygen (MW 32).

The increase in diameter upon Ca^2+^ chelation (Figure 4a,c) resulted from electrostatic repulsion among negatively charged alginate carboxyl groups, leading to water uptake and swelling [45]. This phenomenon is common in alginate-based hydrogels and can be controlled to some extent using Ba^2+^ and alginate with a high proportion of guluronic acid [46]. However, considering that thin membrane and loss of sphericity reduce the biocompatibility of the capsule in vivo [16], using only Ca^2+^ chelators for Ca-alginate microgel liquefaction is inappropriate. For example, EDTA liquefies the microgel more rapidly than citric acid and does not inhibit the survival and function of the encapsulated cells [47]. Thus, the selection of an appropriate chelating agent is important. In contrast to chelation, incomplete liquefaction using alginate lyase without changes in morphology (Figure 4d) indicated that the presence of alginate within the capsule is due to the electrostatic interaction with Ca^2+^ and Chitosan–Ph, and limited diffusion of alginate lyase (Section 4.2 and Section 4.3). Although alginate lyase is cytocompatible with cell-laden microcapsules [48], strategies for promoting its liquefaction are required. In this study, liquefaction by both chelation and enzymatic degradation without swelling (Figure 4e) may occur because degraded alginate cannot form a network and can easily diffuse out of the capsule when Ca^2+^ is chelated.

### 4.2. Effect of Covalent Crosslinking on Microcapsule Stability

Sodium alginate is widely used in microbeads because of its gelability under mild conditions [49,50,51]; however, Ca-alginate beads alone exhibit swelling and insufficient mechanical stability for use in cell therapy, leading to a low success rate [45,52]. Therefore, methods for improving the stability of alginate microgel beads are required.

The osmotic pressure test evaluates microcapsule stability based on deformation resistance and elastic properties and is consistent with the in vivo stability test [53]. In this test, APA microcapsules, which are commonly used in cell therapy, gradually disintegrated over time owing to their weak intermembrane interactions (Figure 5), consistent with previous findings [54]. LBL-coated Chitosan/HA microcapsules and non-crosslinked Chitosan–Ph/HA-Ph microcapsules also could not maintain their morphology due to the relatively weak electrostatic interaction (Figure 5 and Figure S1). In contrast, covalent crosslinking provides greater stability against osmotic pressure (Figure 5), because crosslinking and liquefaction increase the elastic contribution of the capsules relative to their viscous contribution. This is consistent with the crosslinking density (Figure 3) and recovery rates of the crosslinked microcapsules (Figure 6, Table 1). A similar phenomenon was observed in other covalently crosslinked microcapsules using polyethylene glycol, whose crosslinked polymer chains deformed like elastic springs and returned to their original shapes after stress removal [55]. However, excessive H_2_O_2_ deactivates HRP and degrades polymers [56,57], resulting in reduced elasticity of the crosslinked microcapsules (Table 1). This is consistent with macro-scale hydrogels formed via HRP-mediated reactions, in which the crosslinking density and hydrogel pore size can be adjusted with H_2_O_2_ concentration [58]. Therefore, adjusting the H_2_O_2_ concentration is critical for microcapsule stability. This HRP-mediated reaction is considered superior to other systems in that its material properties can be controlled by optimizing the concentrations of HRP, polymer, H_2_O_2_, and Ph content [59].

Crosslinking restricts enzyme diffusion and delays enzymatic degradation [60]. Considering the molecular weights of hyaluronidase (MW 55 kDa) and yatalase (MW 30–110 kDa), the pore size of the crosslinked capsules is likely in the order of tens of kDa or less. Previous studies on the enzymatic degradation of polyion complexes prepared without a crosslinking agent revealed that enzymes adsorb onto alginate/chitosan polyion complexes, but the degradation is inhibited by strong polymer–polymer interactions [61]. Conversely, the HA–chitosan polyion complex protects HA from hydrolysis [62]. Consequently, the stability of the crosslinked microcapsules against the enzymes was attributed to their electrostatic coating and the reduction in pore size by HRP-mediated crosslinking (Figure 7). In addition, because Ph modification can be applied to both natural and synthetic polymers [63], the use of different polymer types may improve the stability of HRP-mediated crosslinked microcapsules against enzymatic degradation. However, in vivo conditions were not simulated because the relevant in vivo enzyme activity remains unclear. Although an in vitro enzymatic stability test was conducted under the conditions that enzymes degraded HA-Ph and Chitosan–Ph macroscale hydrogels within one day and maintained their activity for 7 d, an in vivo stability test is necessary. Furthermore, studies on the storage period and long-term stability after manufacturing are needed.

### 4.3. Limited Permeability of Crosslinked Microcapsules

γ-Globulins are a group of serum proteins containing antibodies such as IgG, a representative small-molecular-weight antibody (MW 150 kDa, hydrodynamic diameter 10.7 nm). Preventing the diffusion of this antibody into microcapsules is required for immune protection. For example, alginate microgel beads do not completely block IgG diffusion, indicating a lack of immune isolation [64,65]. Conversely, coating and crosslinking may have contributed to the limited diffusion of γ-globulin (Figure 8a,b). Considering the stability experiments against enzymes (Figure 7) and the permeability of AF-BSA (Figure 8c,d), the crosslinked microcapsules exhibited size-selective permeability under the present experimental conditions. Based on the restricted diffusion of γ-globulin and the penetration of FITC-BSA, the apparent molecular-weight cut-off of the crosslinked capsules under the present experimental conditions was estimated to be greater than 66 kDa but less than 150 kDa, which is within the range reported for other microcapsules [33]. Photocrosslinked microcapsules with similar size-selective permeability have been reported to achieve blood glucose control in vivo [66]. In the present enzymatically crosslinked system, a similar permeability range may be tuned by increasing the crosslinking density through multilayer coating via LBL assembly. These findings suggest that the crosslinked membrane can reduce penetration of antibody-sized macromolecules under the present in vitro conditions. However, this does not imply complete exclusion of all harmful immune components, because smaller inflammatory mediators may still diffuse across the membrane. Therefore, the present system should be interpreted as providing immunoisolation-related barrier function to some extent, rather than complete immune protection. Co-encapsulation of anti-inflammatory agents may be useful for reducing inflammatory responses associated with such diffusible mediators [67].

Our experiments were limited in that precise molecular weight cut-off values and long-term protective effects were not evaluated. Moreover, the possibility that positively charged proteins adhere to the capsule surface cannot be ruled out [68,69], necessitating in vivo analysis of fibrosis, immune cell adhesion, and cytokine levels.

Although it is necessary to limit the penetration of the immune system by reducing the pore size of the capsules, this must not hinder the diffusion of insulin (MW 5.8 kDa) secreted by the encapsulated β-cells. Crosslinked microcapsules may ensure the free release of insulin (Figure 9), similar to the microcapsules that achieved blood glucose control in diabetic mice [70]. Despite the differences in charge and structure of these molecules, this experiment is consistent with the insulin secretion assay from encapsulated β-cells, as described below.

### 4.4. Cell Encapsulation for β-Cell Transplantation

The cytocompatibility of the fabrication method (Figure 10) was consistent with that of previously reported HRP-mediated hydrogels [71,72]. Considering that β-cells can detoxify micromolar levels of H_2_O_2_ [73], it is possible to avoid impairing cell function or survival by adjusting H_2_O_2_ concentrations. Measurement of oxidative stress markers would further evaluate the effect of H_2_O_2_ exposure on cell survival. Unlike previous crosslinked microcapsule fabrication methods, our method is advantageous because it uses materials with higher cytocompatibility under mild conditions [16,20]. Although it takes longer than one-step encapsulation methods [20], it is simple and does not require sophisticated machinery.

The cytocompatibility of the encapsulated iGL cells (Figure 11) was due to the cell-friendly materials and favorable diffusion of low-molecular-weight substances, such as oxygen and nutrients. Pancreatic cells express cadherins, which are responsible for cell–cell adhesion and cluster formation [74]. Spheroids of INS-1 cells, the precursor of iGL cells, showed improved survival rate, lifespan, and insulin secretion function due to acquiring a tissue-like structure and containing granulated cells with ultrastructure similar to mature β-cells [75]. Therefore, we hypothesized that iGL cells also proliferated while adhering to each other. Similarly, HA-embedded islets exhibited higher survival rates and insulin secretion, along with lower immunogenicity, than alginate-encapsulated islets [76]. Therefore, encapsulation may provide a suitable 3D environment similar to an extracellular matrix for the enclosed β-cells [77,78].

Despite the potential for limited diffusion of insulin (isoelectric point pH 5.4) due to the electrostatic interaction with Chitosan–Ph, the insulin release behavior was similar to that observed in naked cells (Figure 12). This may be because the charge of Chitosan–Ph was partially neutralized by electrostatic interactions with the surrounding HA-Ph (Figure 2), and it exhibited excellent diffusion properties for low-molecular-weight substances such as glucose. Previous studies have reported that diffusion restrictions due to the coating can sometimes result in a decrease in the stimulation index (SI) [79]. Therefore, the parameters controlling the pore size of the capsules are important. Furthermore, SI was equivalent to that of microcapsules covalently bonded using ethylamine-bridged EGCG dimers, demonstrating therapeutic efficacy in a diabetic mouse model [16]. The evaluation of first- and second-phase insulin secretion is needed to investigate the function in detail, as these are necessary to control the blood glucose levels in the early postprandial period and over the long term, respectively. Moreover, SI exceeded 2 depending on the cell type, such as pancreatic or hepatocyte-derived pancreatic islets [80,81]. For treating type 1 diabetes, transplantation of primary islets or islets derived from hepatocytes is more suitable due to their mature insulin secretion function. These islets are susceptible to cell death and loss of function due to ER stress and mitochondrial dysfunction caused by hypoxia and inflammation [82]. In particular, necrosis tends to occur at the center due to restricted oxygen diffusion [83]. Although HRP-crosslinked microcapsules may provide a useful platform with suitable permeability, it is unclear whether this is sufficient for islets.

Crosslinked microcapsules have great potential for cell therapy because of their stability and cytocompatibility. Further functionalization such as through the integration of blood-vessel-like structures around capsules may ensure oxygen and nutrient supply [84]. HA-Ph is expected to promote vascular endothelial cell proliferation and lumen formation [85]. Therefore, the introduction of blood vessels may enhance cell survival and function. Furthermore, the addition of insulin-like growth factor II suppresses β-cell apoptosis [86]. Thus, microcapsules containing both cells and growth factors can enhance cell survival and function.

## 5. Conclusions

Microcapsules with covalently crosslinked LBL membranes composed of Chitosan–Ph and HA-Ph exhibited enhanced stability under osmotic stress and resistance to enzymatic degradation compared with conventional microcapsules, and the membrane substantially reduced the permeation of γ-globulin under in vitro conditions. β-cells encapsulated within the microcapsules maintained high viability and glucose-responsive insulin secretion, indicating that the microcapsule fabrication process was cytocompatible and that the resulting membrane provided barrier properties suitable for cell encapsulation. These findings highlight the potential of microcapsules with enzymatically crosslinked LBL membranes for the development of cell-encapsulation systems.

## Figures and Tables

**Table 1:** Swelling and recovery ratios of each sample.

Concentration of H_2_O_2_ (mM)	0.01	0.1	1
Swelling ratio (−)	1.6 ± 0.1	1.5 ± 0.1	1.4 ± 0.1
Recovery ratio (−)	0.765 ± 0.003	0.847 ± 0.003	0.837 ± 0.003

## Data Availability

The raw data supporting the conclusions of this study will be made available by the authors upon request.

## Supplementary Materials

The following supporting information can be downloaded at: https://www.mdpi.com/article/10.3390/polym18091115/s1, including details of the synthesis procedures for rhodamine-grafted Chitosan–Ph, 5-aminofluorescein-grafted alginate, and FITC-γ-globulin. Figure S1. Intact capsule ratio of HA/Chitosan microcapsules in osmotic pressure tests. Effect of (a) osmotic pressure change with sequential immersion in PBS in ultrapure water (H_2_O) for 2 h and in PBS for 15 min. (b) Effect of incubation time in PBS. The value at day 0 corresponds to the capsules immediately after the sequential immersion test in (a) (n > 170 capsules per preparation, 3 independent preparations; bars: S.D.).

## Abbreviations

The following abbreviations are used in this manuscript:

HA-Ph	Phenol-grafted hyaluronic acid
APA	alginate-poly-L-lysine
Chitosan–Ph	Phenol-grafted Chitosan
RB	Rhodamine B
AF	5-aminofluorescein
HRP	Horseradish peroxidase
BAM	Biocompatible anchor for cell membrane
SI	Stimulation index
CF-KRH	Calcium-free Krebs–Ringer HEPES-buffered solution
PI	Propidium iodide
LBL	Layer-by-layer
BSA	Bovine serum albumin
BSA-KRH	KRH solution (pH 7.4) containing 0.01 *w*/*v*% BSA
G-BSA-KRH	Glucose-containing BSA-KRH
