# Supplementary material for: Enzymatically Crosslinked Chitosan–Hyaluronic Acid Layer-by-Layer Microcapsules with Controlled Permeability and Enhanced Stability for Cell Encapsulation
Source: Polymers (Basel). 2026 Apr 30;18(9):1115. doi: 10.3390/polym18091115 (PMC13165372; doi:10.3390/polym18091115)
Supplement: Supplementary file 1 [file polymers-18-01115-s001.zip › polymers-4254430-supplementary.pdf]

Supplementary Material

Enzymatically Crosslinked Chitosan-Hyaluronic Acid Lay-er-by-Layer Microcapsules with Controlled Permeability and Enhanced Stability for Cell Encapsulation

Ririko Terada[a], Shinji Sakai[*,a]

[a]Department of Materials Engineering Science, Graduate School of Engineering Science, The University of Osaka, Toyonaka, Osaka, 560-8531, Japan.

*Corresponding author: sakai@cheng.es.osaka-u.ac.jp

Synthesis of Rhodamine-Grafted Chitosan-Ph (RB-Chitosan-Ph)

NHS-Rhodamine was synthesized using previously reported methods [1]. Briefly, 5 mM rhodamine B and 5 mM N-hydroxysuccinimide (NHS)were added to acetonitrile and stirred in a water bath at 45°C. Acetonitrile containing 1 mM N, N'-Dicyclohexylcarbodiimide was added to the mixture. After stirring in the water bath for 1 h, the reaction solution was stirred at room temperature for 20 h. Vacuum filtration and evaporation were performed to remove byproducts and acetonitrile. The product was stored at 4 °C.

Phenol grafted Chitosan (Chitosan-Ph) was dissolved in PBS (pH 6.0) at 3.0% (w/v), and then, N, N-Dimethylformamide containing 3.0% (w/v) NHS-Rhodamine was added in a 2:1 ratio by volume. After stirring at room temperature for 22 h, the reaction solution was dialyzed against deionized water and freeze-dried.

Synthesis of FITC-Grafted HA-Ph

Hyaluronic acid (Na-HA) was dissolved in 10 mM MES buffer at 0.75% (w/v), and the pH was adjusted to 6.0. 0.5% (w/v) FITC-I, 0.525% (w/v) water-soluble carbodiimide (EDC), 0.314% (w/v) NHS, and 1.21% (w/v) tyramine were added sequentially. After stirring at room temperature overnight, the mixture was dialyzed against deionized water and then freeze-dried.

Synthesis of 5-AF-Grafted Alginate (AF-Alginate)

Sodium alginate was dissolved in 10 mM MES buffer at 2.0% (w/v) and the pH was adjusted to 6.0. 0.7% (w/v) EDC, 0.2% (w/v) NHS, and 0.06% (w/v) 5-aminofluorescein (AF) were sequentially added. After stirring for 20 h at room temperature, the product was precipitated with acetone, washed with 80% ethanol and 100% ethanol, and finally dried under vacuum.

Synthesis of FITC-Grafted γ-globulin (FITC-γ-globulin)

DMSO containing 0.1% (w/v) of FITC-I was added to a 0.1 M Na_2CO_3 solution (pH 9.5) containing 0.1% (w/v) γ-globulin in an ice bath in a 1:10 ratio by volume. After stirring overnight at 4°C in the dark, the reaction solution was dialyzed against PBS (pH 7.4) and stored at 4°C in the dark.

Osmotic pressure test of HA/Chitosan microcapsules

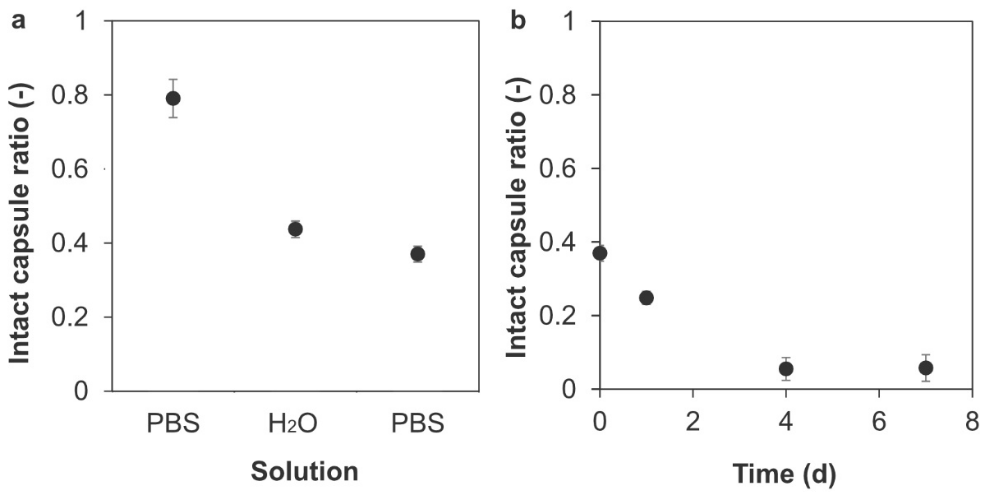

Figure S1. Intact capsule ratio of HA/Chitosan microcapsules in osmotic pressure tests. Effect of (a) osmotic pressure change with sequential immersion in PBS in ultrapure water (H_2O) for 2 h and in PBS for 15 min. (b) Effect of incubation time in PBS. The value at day 0 corresponds to the capsules immediately after the sequential immersion test in (a) (n > 170 capsules per preparation, 3 independent preparations; bars: S.D.).

Reference

1. Meng, Q.; Yu, M.; Zhang, H.; Ren, J.; Huang, D. Synthesis and Application of N-Hydroxysuccinimidyl Rhodamine B Ester as an Amine-Reactive Fluorescent Probe. *Dyes and Pigments* **2007**, *73*, 254–260, doi:10.1016/j.dyepig.2005.12.008.
